# Serum Golgi protein 73 (GP73) is a diagnostic and prognostic marker of hepatocellular carcinoma

**DOI:** 10.3389/fmed.2025.1571761

**Published:** 2025-04-14

**Authors:** Qian Wang, Hongxia Cui, Yaping Zhu, Yichun Zhu, Chunyan He

**Affiliations:** ^1^Department of Clinical Laboratory, The Second Affiliated Hospital of Soochow University, Suzhou, Jiangsu, China; ^2^Department of Pathology, The Second Affiliated Hospital of Soochow University, Suzhou, Jiangsu, China; ^3^Department of Clinical Laboratory, Kunshan Hospital of Chinese Medicine, Kunshan, Jiangsu, China

**Keywords:** Golgi protein-73 (GP73), alpha-fetoprotein (AFP), hepatocellular carcinoma, diagnostic marker, tumor immune microenvironment

## Abstract

**Introduction:**

Hepatocellular carcinoma (HCC) constitutes a significant global health burden, and is characterized by limited early detection methods and poor survival rates. Golgi protein 73 (GP73), previously associated with liver-related diseases, has a controversial diagnostic value for HCC. The present study aimed to determine the diagnostic efficacy of serum GP73 (sGP73) levels in HCC and to explore their potential correlations with the development of HCC.

**Methods:**

The levels of sGP73 and serum alpha-fetoprotein (sAFP) were measured in 134 HCC patients, 200 healthy controls (HCs), and 45 non-HCC patients with various liver diseases. Additionally, immunohistochemical staining was conducted on paraffin-embedded tissue samples obtained from 30 HCC patients to examine the expression of CD4^+^ T cells, CD8^+^ T cells, Foxp3^+^ Treg cells, Ki-67, and interferon-gamma (IFN-γ) in the tissue specimens.

**Results:**

sGP73 and sAFP were markedly higher in HCC patients than in HCs and non-HCC patients. However, sGP73 showed significantly higher sensitivity as a diagnostic marker for HCC than sAFP. The combination of sGP73 and sAFP further improved the accuracy (AUROC: 0.830). Besides, in the immunohistochemical staining analyses, sGP73-positive patients had lower expression of CD4^+^ and CD8^+^ T cells, higher expression of Foxp3^+^ Treg cells, higher expression of nuclear Ki67, and lower expression of IFN-γ than GP73-negative patients. In addition, sGP73-positive patients tended to have higher mortality rate, higher rate of metastasis, higher AFP levels, and more pronounced liver inflammation and damage than GP73-negative patients.

**Conclusions:**

sGP73 could be utilized as a marker for the diagnosis of HCC, and may be implicated in the development of HCC through its interactions with the tumor microenvironment.

## Introduction

Hepatocellular carcinoma (HCC) accounts for more than 80% of newly diagnosed cases of primary liver cancer, and is the third leading cause of cancer-related deaths worldwide (1). Common risk factors associated with HCC include chronic infection with hepatitis B virus or hepatitis C virus, alcohol abuse, liver cirrhosis (LC), and metabolic diseases such as non-alcoholic fatty liver disease (NAFLD). While surgical resection or liver transplantation is deemed effective for treatment of HCC, the 5-year mortality rate remains >88% (2, 3). Unfortunately, the majority of HCC cases are diagnosed at advanced stages, rendering curative treatments inappropriate (4). Among the current screening methods, serum alpha-fetoprotein (sAFP) levels and abdominal ultrasound are widely used for HCC diagnosis and surveillance (5). However, ultrasound is operator-dependent and can be influenced by the operator's skills and experience as well as the patient's obesity level. The sensitivity of screening using ultrasound alone or combined with sAFP was reported to be 47 and 63%, respectively, for early-stage HCC (6). Imaging studies can only detect tumor nodules of a certain diameter, typically at an advanced stage. Meanwhile, sAFP is not an ideal screening test due to its low sensitivity (<50%) and the fact that elevated AFP levels can be observed in acute liver diseases and pregnancy (7, 8). Therefore, the development of more sensitive and specific tumor biomarkers for early detection of HCC in the at-risk population will contribute to enhanced clinical management and improved survival outcomes.

Golgi protein 73 (GP73) is a Golgi membrane protein involved in protein trafficking and glycosylation processes. Recent studies have highlighted its relevance in various diseases, including liver-related conditions. For example, immunohistochemical studies demonstrated consistent expression of GP73 in biliary epithelial cells of the stomach, intestines, and prostate, while hepatocytes in the normal liver exhibited no expression (9, 10). However, GP73 expression was strongly upregulated in hepatocytes from patients with liver diseases (10). GP73 expression may contribute to Golgi apparatus integrity, but its precise biological role is uncertain. Its levels may reflect microenvironmental alterations or indicate distinct biology in liver disease cells (11). In 2005, Block et al. reported for the first time that in animal liver cancer cells GP73 is highly expressed, and in human patients with HCC the serum level of GP73 is significantly increased (12). In recent years, a growing body of research has explored the potential diagnostic significance of sGP73 in HCC, but the findings have remained controversial (13, 14). Nevertheless, immune escape is a pivotal characteristic of HCC, and there is evidence suggesting that the tumor immune microenvironment plays a crucial role in driving tumor initiation, progression, and metastasis (15, 16). An early study has reported that GP73 facilitates cancer metastasis as well as proliferation, and GP73 upregulates MMP-7 and CD44, the factors highly expressed in metastatic cancer cells (17). Therefore, it is deemed that GP73 might promote EMT of cancer cells through upregulating the levels of EMT-related proteins. Also, two recent studies have reported that GP73 upregulates programmed cell death ligand-1 (PD-L1) and facilitates immune escape of HCC cells through activating EGFR signaling pathway, which prove that, similar to the former study, GP73 also plays key roles in immune microenvironment (18, 19). However, the regulatory mechanisms are unclear. Therefore, the associations between sGP73 levels and the tumor immune microenvironment of HCC remains to be studied. Recent studies have recognized liver-infiltrating immune cells, including regulatory T (Treg) cells and effector T cells (such as CD4^+^ T cells and CD8^+^ T cells), as potential prognostic factors in HCC patients (20–22). On the one hand, Treg cells, identified by expression of Foxp3, play critical roles in regulating immune responses and promoting immune tolerance in cancer (23–25). On the other hand, liver-infiltrating lymphocytes, including CD8^+^ T cells, have the potential to function as effector cells in regulating tumor expansion and operate optimally by relying on CD4^+^ T helper 1 immune responses. To date, however, the underlying mechanisms that link GP73 and development of HCC remain poorly understood. In the present study, we aimed to determine the diagnostic utility of sGP73 levels in HCC. In addition, by analyzing the sGP73 levels alongside clinicopathological findings in HCC patients, we examined the possible correlations between sGP73 and the tumor immune microenvironment of HCC.

## Materials and methods

### Study population

A total of 179 patients were enrolled from June 2021 to July 2022. Among these patients were 134 newly diagnosed cases of HCC, 15 cases of LC, 15 cases of NAFLD, and 15 cases of chronic hepatitis B (CHB) without liver cirrhosis. The diagnosis of HCC was based on pathological and/or radiological findings (26). LC diagnosis was confirmed using available data, including radiological, laboratory, and clinical assessments. The diagnosis of NAFLD relied on abdominal ultrasonography. CHB was characterized by persistent HBsAg positivity for at least 6 months. Simultaneously, 200 healthy controls (HCs) without any noticeable diseases or infections were recruited from the health check-up center at our hospital. The inclusion criteria were: (1) no prior receipt of anticancer treatments, such as surgery, radiotherapy, chemotherapy, immunotherapy, or ablation; (2) no missing data; (3) first admission to hospital only. The exclusion criteria were: (1) age <18 years; (2) history of other malignant tumors. Tumor staging was determined by the United Network of Organ Sharing-modified TNM staging system for HCC (27).

The study was approved by the Second Affiliated Hospital of Soochow University's Institutional Review Board (Approval No. LC2024008-I01). All methods were performed in accordance with the relevant guidelines and regulations of the Ethics Committee of the Second Affiliated Hospital of Soochow University.

### Data collection and blood sample processing

Demographic information, clinical variables, and laboratory data were obtained from medical records. The sAFP and sGP73 levels were evaluated in serum samples that had been stored at −80°C.

### Analysis techniques

sGP73 levels were detected by ELISA (Hotgen Biotech Inc., Beijing, China) and quantified in accordance with the manufacturer's protocol. sAFP levels were measured using a Cobas E801 biochemical immunoanalyzer (Roche Diagnostics, Mannheim, Germany).

### Follow-up evaluation

The follow-up period commenced from the date of liver resection. The endpoint was survival at 6 months.

### Immunohistochemical analysis

Immunohistochemical staining was performed on paraffin-embedded tissue samples obtained from 30 HCC patients. Sections of the liver tissues were dewaxed, rehydrated, subjected to antigen retrieval, and incubated with primary antibodies at 4°C overnight. Color development was achieved using diaminobenzidine as the substrate, following by counterstaining with hematoxylin. Finally, the slides were mounted with neutral gum and transported for central reading. The immunohistochemical findings for each marker were evaluated by two senior pathologists under double-blind conditions. The antibodies used were: anti-CD4 (1:50; Zhongshan Golden Bridge Biotech Co., Beijing, China), anti-CD8 (1:400; 85,336 S; Cell Signaling Technology, Danvers, MA), anti-Ki67 (1:20; MXB Biotechnologies Inc., Fuzhou, China), anti-Foxp3 (1:100; Abcam plc, Cambridge, UK), and anti-IFN-γ (1:100; Abcam plc).

### Immunostaining scoring system

Immunostaining of CD4, CD8, Foxp3, Ki67, and IFN-γ in the liver tissues was assessed using a scoring system that encompassed staining intensity [0 = none (negative), 1 = weak (light yellow), 2 = moderate (brown-yellow), 3 = strong (dark brown)] and proportion of expressing cells (0 = 0%, 1 = 1–25%, 2 = 26–50%, 3 = 51–75%, 4 = 76–100%). After multiplying the two scores together, the final scores ranged from 0 to 12.

### Statistical analysis

Statistical analyses were conducted using SPSS ver. 19 or MedCalc ver. 19.7. Values of *P* < 0.05 were considered statistically significant. Continuous and categorical variables were expressed as median [interquartile range (IQR)] and number, respectively. The Mann–Whitney or chi-square test was used to compare the differences between two groups. Receiver operator characteristic (ROC) curve analyses were conducted and comparisons were done by the DeLong test to assess the GP73 and/or AFP for the diagnosis of HCC. The obtained area under the ROC curve (AUC) values were used to estimate diagnostic values of sGP73 and sAFP levels in HCC.

## Results

### Study subjects

**Figure 1:**
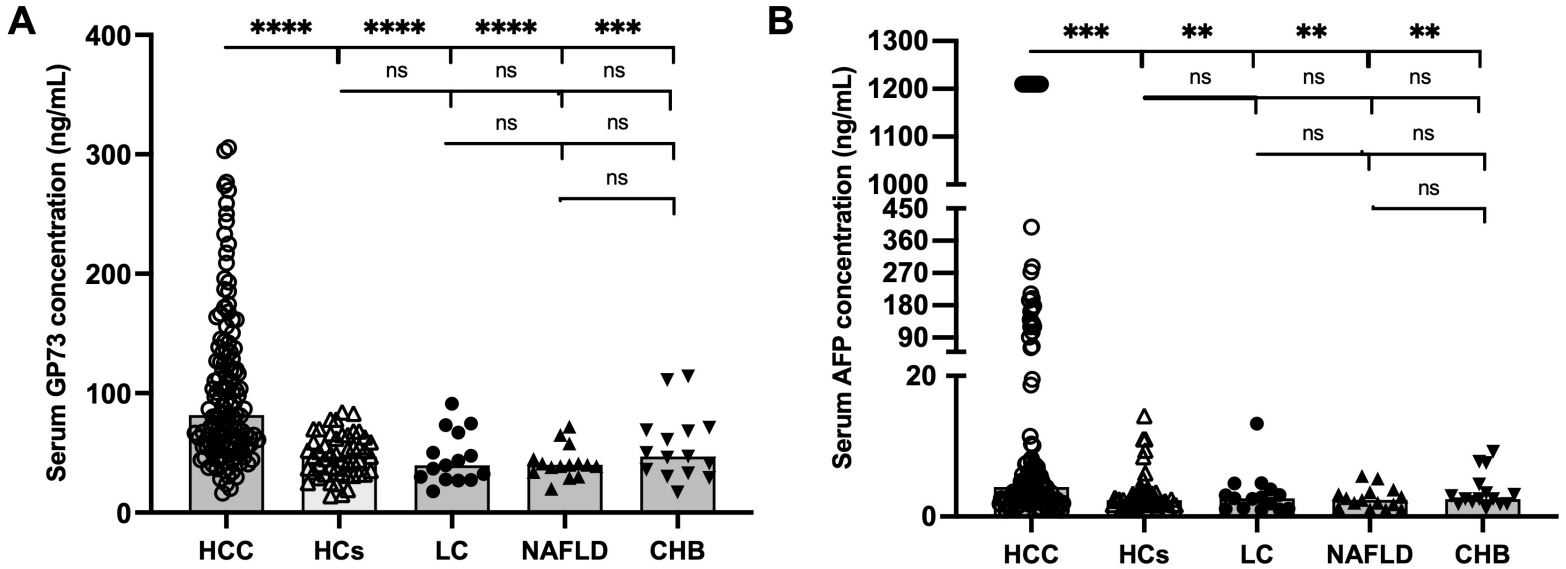
Levels of sGP73 and sAFP in the HCC and non-HCC patient groups. **(A)** Comparisons of sGP73 levels among the HCC patients, HCs, LC patients, NAFLD patients, and CHB patients. **(B)** Comparisons of sAFP levels among the HCC patients, HCs, LC patients, NAFLD patients, and CHB patients. The three horizontal bars in each group represent the median value and the upper and lower range values. **P < 0.01, ***P < 0.001, ****P < 0.0001, ns, non-significant; HCC, hepatocellular carcinoma; HCs, healthy controls; LC, liver cirrhosis; NAFLD, Non-alcoholic fatty liver disease; CHB, chronic hepatitis B.

A total of 229 subjects were included. The demographic and clinical characteristics of the participants are outlined in Table 1. The sGP73 and sAFP levels were higher in HCC patients than in HCs, LC patients, NAFLD patients, and CHB patients. However, the sGP73 and sAFP levels did not differ significantly among the patients with each liver disease (Figure 1).

**Table 1:** Clinical characteristics of the study subjects.

**Variables**	**HCC (*n* = 134)**	**HCs (*n* = 50)**	**LC (*n* = 15)**	**NAFLD (*n* = 15)**	**CHB (*n* = 15)**	***P^1^***
Age (year)	62.1 ± 11.3	56.8 ± 12.9	66.2 ± 10.9	56.1 ± 9.9	55.7 ± 10.4	0.429
Gender (M/F)	98/36	33/17	9/6	10/5	9/6	0.653
Alanineamino transferase (U/L)	41.0 (20.8–70.5)	19.0 (14.0–28.0)	14.0 (11.0–20.0)	27.0 (17.0–31.0)	26.0 (18.0–56.0)	<0.001
Aspartateamino transferase (U/L)	32.5 (22.0–56.0)	26.5 (19.0–60.3)	18.0 (15.0–25.0)	20.0 (16.0–25.0)	31.0 (23.0–52.0)	<0.001
Alkaline phosphatase (U/L)	127.0 (88.0–245.8)	64.5 (52.0–83.0)	86.0 (70.0–136.0)	66.0 (57.0–86.0)	82.0 (69.0–94.0)	<0.001
Gammaglutamyl transferase (U/L)	86.0 (35.8–202.5)	21.5 (15.0–32.5)	20.0 (12.0–27.0)	30.0 (14.0–45.0)	26.0 (18.0–44.0)	<0.001
Albumin (g/L)	39.1 (33.1–44.4)	49.7 (47.2–50.6)	43.9 (34.0–45.1)	48.0 (46.0–50.0)	46.2 (39.6–50.1)	<0.001
Total bilirubin (μmol/L)	18.7 (12.2–38.0)	10.8 (8.9–16.2)	9.4 (6.3–12.7)	11.5 (9.0–16.9)	17.0 (8.3–19.0)	<0.001
AFP (ng/mL)	4.2 (2.4–40.9)	2.3 (1.7–3.3)	2.6 (1.1–3.9)	2.4 (1.2–3.5)	2.5 (1.8–4.6)	<0.001
GP73 (ng/mL)	81.6 (56.6–134.2)	47.0 (32.8–62.3)	39.6 (27.8–67.0)	40.0 (34.0–45.0)	47.0 (33.0–69.0)	<0.001

Data are expressed as number, mean ± SD, or median (range).

HCC, hepatocellular carcinoma; HCs, healthy controls; LC, liver cirrhosis; NAFLD, Non-alcoholic fatty liver disease; CHB, chronic hepatitis B, AFP, alpha-fetoprotein; GP73, Golgi Protein 73. *P*^1^ Difference among the 4 groups.

### Diagnostic performance of sGP73 and sAFP levels for HCC

**Figure 2:**
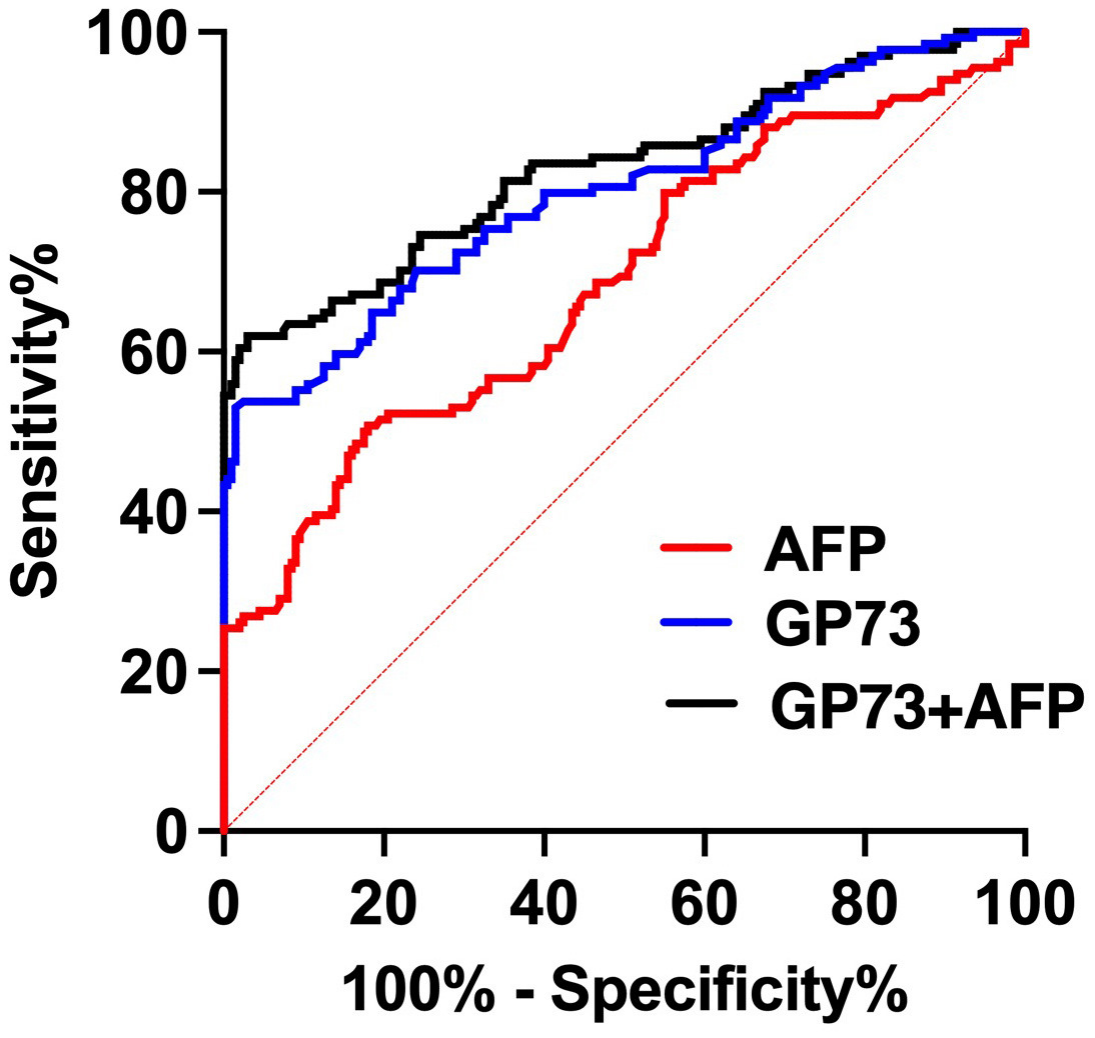
ROC curves of GP73 and/or AFP for the diagnosis of HCC.

We collected 134 patients with HCC and 200 healthy subjects, conducted a receiver operating characteristic curve (ROC) analysis and compared by DeLong test to evaluate the abilities of sGP73 and sAFP levels to distinguish between HCC patients and patients with other underlying liver diseases as well as HCs. The optimal cutoff value of sGP73 for HCC diagnosis was 75 ng/mL, with sensitivity of 53.7%, specificity of 93.5%, positive predictive value of 84.7%, and negative predictive value of 75.1%. For sAFP, the optimal cutoff value was 7.1 ng/mL, with sensitivity of 33.6%, specificity of 91.0%, positive predictive value of 71.4%, and negative predictive value of 67.2% (Table 2). These findings indicated that sGP73 served as a more sensitive diagnostic marker for HCC than sAFP. Meanwhile, the diagnostic efficacy of sGP73 for HCC was remarkably higher than that of sAFP, as indicated by the area under the ROC curve (0.800 for GP73 vs. 0.686 for AFP, *P*=0.0034; Figure 2). On the basis of the obtained sGP73 and sAFP cutoff values, 30 of 45 patients with sAFP >7.1 ng/mL had sGP73 >75 ng/mL and 42 of 89 with sAFP ≤ 7.1 ng/mL had sGP73 >75 ng/mL, indicating the potential diagnostic value of sGP73 for diagnosing HCC, particularly in patients with normal sAFP levels (Table 2). Furthermore, when sGP73 and sAFP were combined, the sensitivity and specificity reached 64.9 and 93.0%, respectively, and the AUC increased to 0.830 (Table 3).

**Table 2:** HCC patients with sAFP and sGP73 levels above and below the cutoff values defined by the ROC analyses.

**Parameters**	**GP73>75 (ng/mL)**	**GP73 ≤ 75 (ng/mL)**	**Total (*n*)**
AFP>7.1 (ng/mL)	30	15	45
AFP ≤ 7.1 (ng/mL)	42	47	89
Total *(n)*	72	62	134

AFP, alpha-fetoprotein; GP73, Golgi Protein 73.

**Table 3:** Diagnostic values of sGP73 and sAFP levels in HCC.

**Parameters**	**Sensitivity**	**Specificity**	**AUC**	**95% CI**
GP73	53.7%	93.5%	0.800	0.749–0.850
AFP	33.6%	91.0%	0.686	0.626–0.745
GP73+AFP	64.9%	93.0%	0.830	0.781–0.878

AFP, alpha-fetoprotein; GP73, Golgi Protein 73.

### Associations between sGP73 levels and clinicopathological features in HCC patients

**Figure 3:**
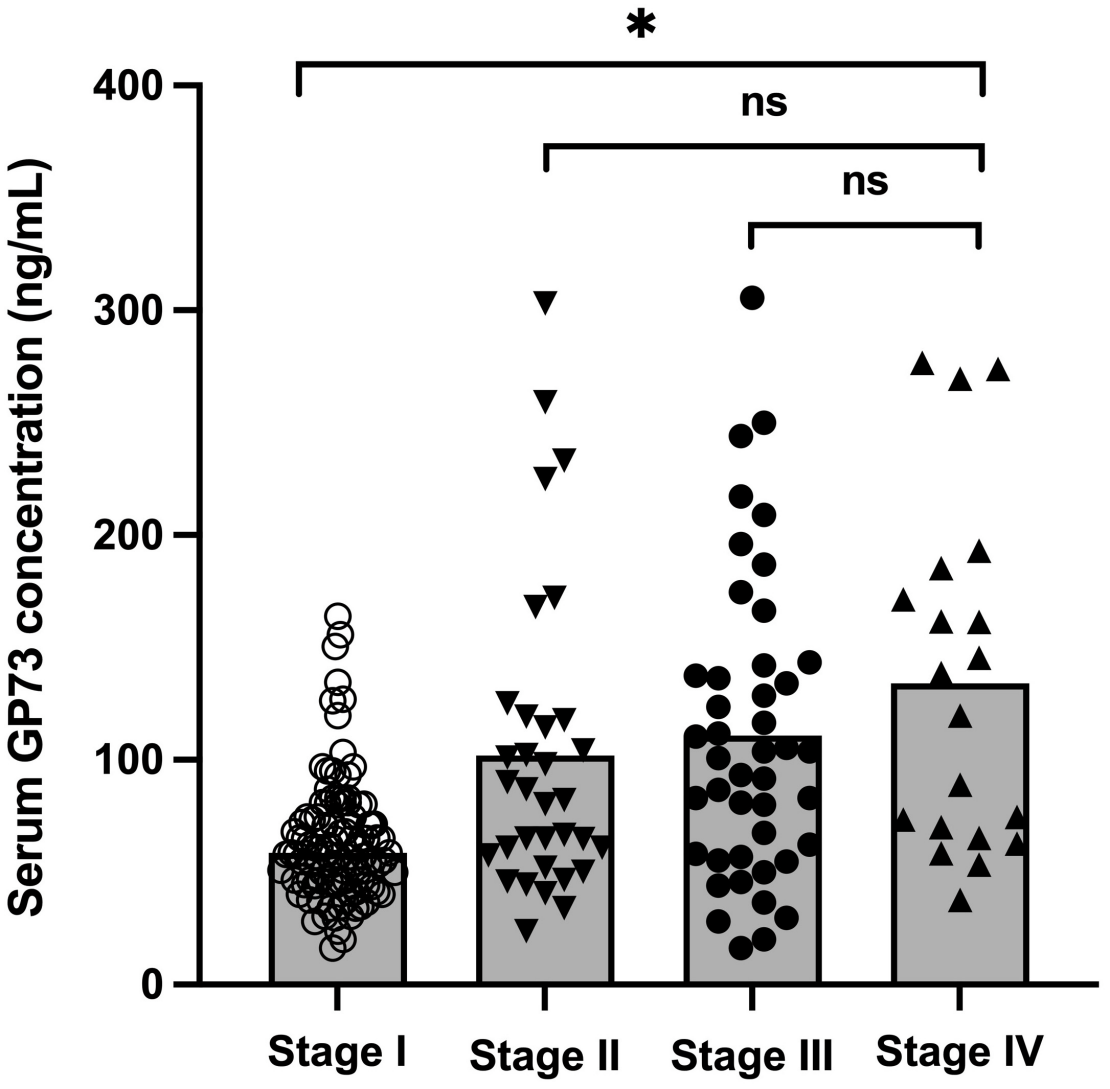
Levels of sGP73 in HCC patients with different clinical stages. The three horizontal bars in each group represent the median value and the upper and lower range values. *P < 0.05, ns, non-significant (*P* > 0.05).

**Figure 4:**
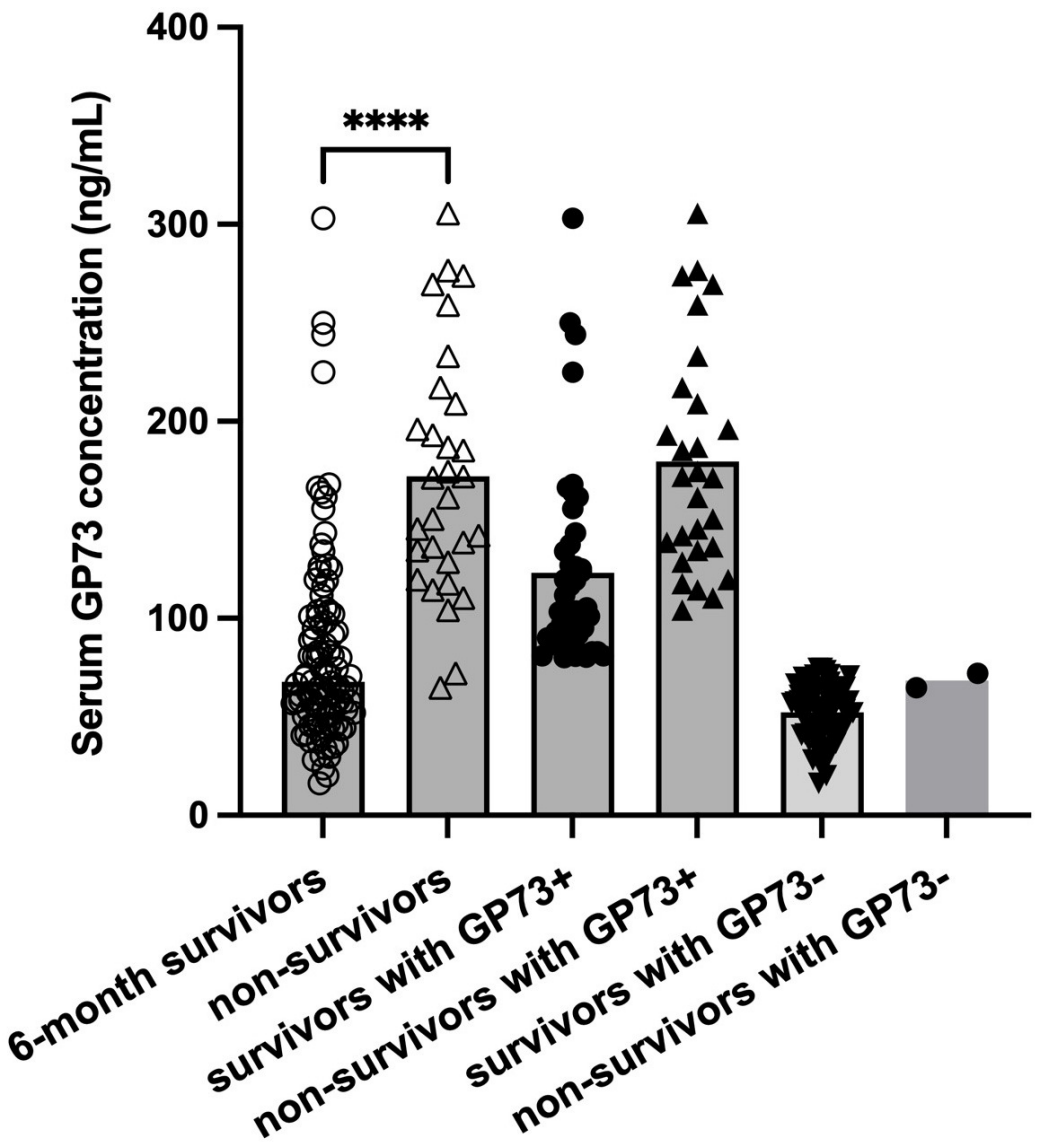
Levels of sGP73 in 6-month survivor and non-survivor group. The three horizontal bars in each group represent the median value and the upper and lower range values. ****P < 0.0001.

According to the sGP73 cutoff value (75 ng/mL), the HCC patients were divided into a GP73-positive group (GP73 >75 ng/mL; *n* = 72) and a GP73-negative group (GP73 ≤ 75 ng/mL; *n* = 62), and the important clinicopathological parameters, such as liver function, sAFP level, tumor differentiation degree, metastasis, mode of treatment, and 6-month mortality after resection, were compared between the two groups. For patients with different clinical stages of liver cancer, the level of sGP73 in stage IV patients was significantly higher than that in stage I (*P* < 0.05; Figure 3). In the sGP73-positive group, only 15 (20.8%) patients received surgical resection for curative purposes, while 57 (79.2%) received TACE (transcatheter arterial chemoembolization) for disease control purposes. Meanwhile, in the sGP73-negative group, 33 (53.2%) patients received surgical resection and 29 (46.8%) received TACE. In addition, sGP73-positive patients tended to have higher mortality rate, higher rate of metastasis, higher AFP levels, and more pronounced liver inflammation and damage than GP73-negative patients (Tables 4, 5). However, there were no significant differences in age, sex, TNM staging, and tumor cell differentiation between the two groups (all *P* > 0.05). The level of sGP73 was significantly higher in non-survivors, compared with that in 6-month survivors (*P* < 0.0001). Furthermore, non-survivors showed increased sGP73 in the GP73-positive group (*P* < 0.05; Figure 4).

**Table 4:** Clinical characteristics of the GP73-positive and GP73-negative patients.

**Variables**	**GP73>75 (ng/mL) (*n* = 72)**	**GP73 ≤ 75 (ng/mL)** **(*n* = 62)**	***P^1^***
Age (year)	61.9 ± 12.2	62.5 ± 10.3	0.942
Gender (M/F)	52/20	46/16	0.797
Alanineamino transferase (U/L)	43.0 (24.3–69.8)	38.0 (19.0–72.3)	0.339
Aspartateamino transferase (U/L)	39.0 (25.3–64.0)	28.0 (20.8–45.3)	0.005
Alkaline phosphatase (U/L)	158.0 (101.0–313.8)	97.0 (71.0–151.5)	0.002
Gammaglutamyl transferase (U/L)	141.0 (56.3–292.3)	50.0 (26.0–127.5)	<0.001
Albumin (g/L)	38.2 (33.0–43.7)	40.6 (33.7–44.4)	0.487
Total bilirubin (μ mol/L)	24.5 (13.0–50.9)	15.8 (11.5–28.8)	0.018
AFP (ng/mL)	5.17 (2.48–120.8)	2.93 (2.32–6.76)	0.044
**TNM staging**, ***n*** **(%)**			0.072
1	15 (20.8%)	25 (40.3%)	
2	18 (25.0%)	15 (24.2%)	
3	27 (37.5%)	14 (22.6%)	
4	12 (16.7%)	8 (12.9%)	
**Differentiation**, ***n*** **(%)**			0.493
High or medium	35 (48.6%)	34 (54.8%)	
Low	37 (51.4%)	28 (45.2%)	
**Treatment modality**, ***n*** **(%)**			<0.001
Surgical resection	15 (20.8%)	33 (53.2%)	
TACE	57 (79.2%)	29 (46.8%)	
**Lymph node metastasis**, ***n*** **(%)**			<0.001
Yes	53 (73.6%)	26 (41.9%)	
No	19 (26.4%)	36 (58.1%)	
**6-month survival**, ***n*** **(%)**			<0.001
Yes	44 (61.1%)	60 (96.8%)	
No	28 (38.9%)	2 (3.2%)	

Data are expressed as number, mean ± SD or median (range).

AFP, alpha-fetoprotein; sGP73, serum Golgi Protein 73.

**P**^**1**^: Difference between the 2 groups.

**Table 5:** Multivariate logistic regression analysis of risk factors associated with GP73 in HCC patients.

**Variables**	**B**	**Wald**	**OR with CI**	***P***
Age (year)	−0.04	2.66	0.961 (0.915–1.007)	0.103
Gender (M/F)	−0.57	1.06	0.565 (0.187–1.675)	0.303
Alanineamino transferase	0.00	0.85	0.997 (0.991–1.004)	0.356
Aspartateamino transferase	0.03	9.66	1.028 (1.012–1.047)	0.002
Alkaline phosphatase	0.00	0.37	1.001 (0.998–1.005)	0.544
Gammaglutamyl transferase	0.00	2.72	1.004 (0.999–1.009)	0.099
Albumin	0.00	0.00	1.001 (0.938–1.07)	0.986
Total bilirubin	0.00	0.08	0.999 (0.992–1.009)	0.781
AFP	0.00	0.45	0.999 (0.998–1.001)	0.503
TNM staging	0.15	0.18	1.161 (0.574–2.369)	0.675
Differentiation (High or medium)	−1.33	1.83	0.263 (0.036–1.769)	0.176
Differentiation (Low)	−0.25	0.10	0.778 (0.165–3.704)	0.748
Treatment modality	1.06	4.14	2.882 (1.058–8.269)	0.041
Lymph Node Metastasis	1.37	4.34	3.922 (1.117–15.096)	0.037
6-month survival	−3.22	12.86	0.04 (0.005–0.191)	<0.001

### Immunohistochemical findings in GP73-positive and GP73-negative HCC patients

**Figure 5:**
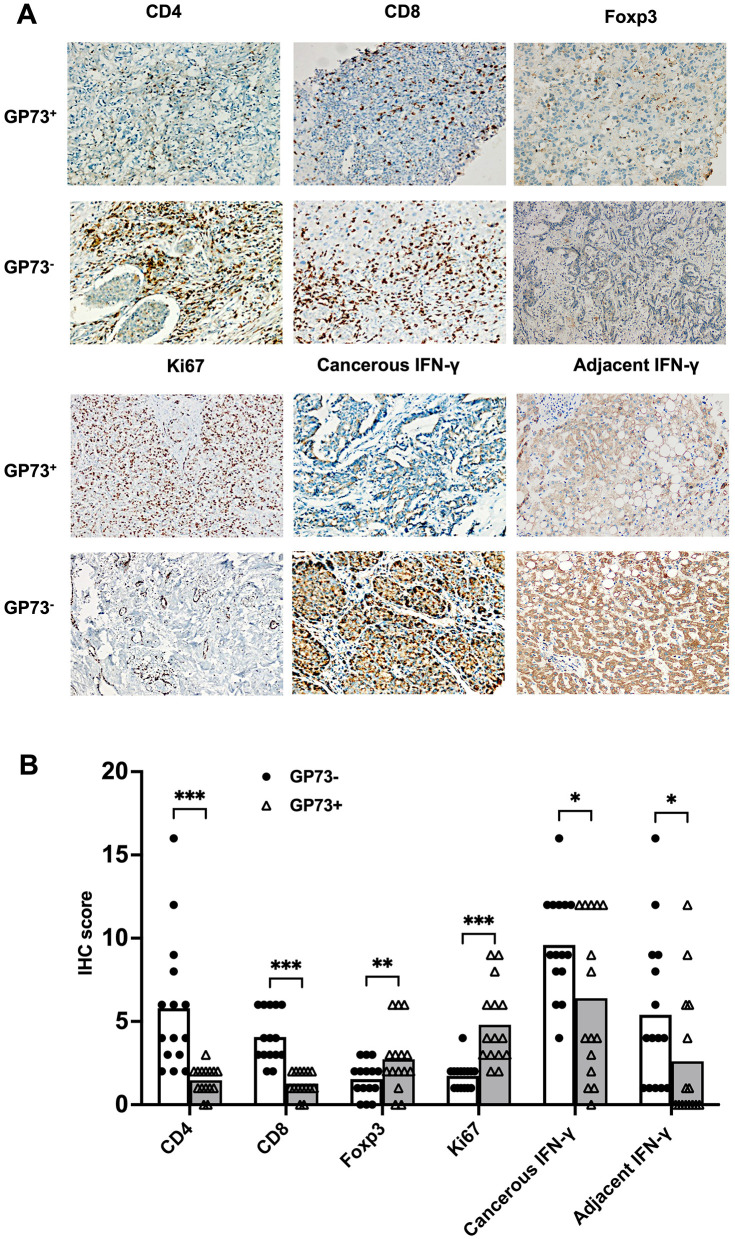
**(A)** Representative immunohistochemical staining of CD4^+^ T cells, CD8^+^ T cells, Foxp3^+^ Treg cells, Ki67, and IFN-γ (cancer tissues and adjacent tissues) in samples from the GP73-positive and GP73-negative HCC patients (200×). **(B)** Comparisons of the expression levels of CD4^+^ T cells, CD8^+^ T cells, Foxp3^+^ Treg cells, Ki67, and IFN-γ (cancerous and adjacent tissues) between samples from the GP73-positive and GP73-negative HCC patients. *P < 0.05, **P < 0.01, ***P < 0.001.

To elucidate the correlations between sGP73 levels and tumor immune microenvironment factors in hepatic tissue, we selected 15 GP73-positive and 15 GP73-negative HCC patients matched for age and sex and conducted hepatic tissue immunohistochemical analyses. Liver-infiltrating immune cells, such as CD4^+^ T cells, CD8^+^ T cells, and Foxp3^+^ Treg cells, and expression levels of Ki67 and IFN-γ were detected by immunohistochemical staining. GP73-positive HCC patients tended to exhibit lower immunohistochemical scores for CD4^+^ T cells and CD8^+^ T cells, higher immunohistochemical score for Foxp3^+^ Treg cells, higher immunohistochemical score for nuclear Ki67, and lower immunohistochemical score for IFN-γ compared with GP73-negative patients (regardless of analysis in cancerous or adjacent tissues). Representative expressions of these markers are shown in Figure 5. In the correlation analyses, sGP73 was negatively correlated with CD4^+^ T cells (*r* = −0.567, *P* = 0.001), CD8^+^ T cells (*r* = −0.662, *P* < 0.001), and IFN-γ level (*r* = −0.409, *P* = 0.025 in cancerous tissue; *r* = −0.368, *P* = 0.045 in adjacent tissue), positively correlated with nuclear Ki67 (*r* = 0.473, *P* = 0.008), and not correlated with Foxp3^+^ Treg cells. In addition, Foxp3^+^ Treg cells were positively correlated with nuclear Ki67 (*r* = 0.325, *P* = 0.047) and negatively correlated with IFN-γ (*r* = −0.595, *P* = 0.001 in cancerous tissue; *r* = −0.5174, *P* = 0.003 in adjacent tissue).

## Discussion

HCC ranks as the third leading cause of cancer-related deaths globally, and is characterized by subtle onset, rapid progression, and unfavorable prognosis. Early identification of liver cancer is crucial for enhancing treatment effectiveness and survival outcomes. Many studies have suggested that sGP73 can serve as a diagnostic marker for HCC, but the results have remained controversial. Our data indicate a significant elevation of sGP73 levels among HCC patients compared with both HCs and non-HCC patients. Meanwhile, sGP73 levels did not differ significantly among patients with each liver disease examined. Nevertheless, a study by Tian and colleagues indicated that sGP73 levels were higher in cirrhotic patients compared with liver cancer and hepatitis patients (28). This variation may be attributed to differences in the study populations and sample sizes. For example, the present study had 15 cases with LC, while Tian and colleagues reported 95 LC cases. Importantly, their cases were more likely to exhibit severe conditions, with higher levels of alanine aminotransferase, aspartate aminotransferase, and total bilirubin, than our cases. Our study also showed that sGP73 levels were not correlated with tumor differentiation, consistent with the findings of Mao et al. (29). In addition, sGP73 exhibited significantly higher sensitivity than sAFP (53.7 vs. 33.6%) as a diagnostic marker for HCC. This difference may be attributed to the possibility that sAFP is directly derived from tumor cells and its levels may be affected by tumor number or size. GP73 is a known Golgi membrane protein, and abnormal function of specific membrane proteins is likely to be associated with the occurrence and development of tumors, irrespective of their size or number. Moreover, among patients with sAFP levels <7.1 ng/mL (based on the cutoff value from the present ROC curve analysis), 47.2% (42/89) had significantly elevated sGP73 levels. This implies that the utility of sGP73 becomes more significant in situations where sAFP levels do not exhibit a significant increase or fail to rise. Consequently, sGP73 may function as a specific and sensitive marker for sAFP-negative HCC. In addition, the sensitivities of sGP73 and sAFP for HCC were 53.7 and 33.6%, respectively. However, their combined detection sensitivity reached 64.9%, indicating that their combined detection could prevent false-negative diagnoses by sAFP alone and significantly improve the overall detection rate.

Immunological mechanisms play a crucial role in malignancy surveillance and control of tumor progression. Accumulating evidence indicates that the tumor immune microenvironment is pivotal in the development of HCC. Specifically, immune dysfunction plays an important role in the onset and progression of HCC (30). However, the associations between sGP73 levels and the tumor immune microenvironment of HCC remain incompletely understood. To date, there has been a general acknowledgment that liver-infiltrating T cells, particularly CD8^+^ T cells, are pivotal in regulating tumor progression. For example, effective CD8^+^ T cells engaged in cytotoxic killing may have a crucial role in the antitumor immune response, releasing granules like perforin and granzymes (31). Consequently, liver infiltrating CD8^+^ T cells have been considered a favorable prognostic indicator in various types of tumors. Previous studies have highlighted a role of Foxp3^+^ Treg cells in regulating tumor immunity, with increased levels of Treg cells observed among tumor-infiltrating lymphocytes in non-small cell lung, ovarian, breast, and pancreatic cancers (32). Through immunohistochemical staining, we observed that GP73-positive patients exhibited higher expression of Foxp3^+^ Treg cells, along with significantly lower levels of CD8^+^ T cells and CD4^+^ T cells compared with GP73-negative patients. Furthermore, sGP73 levels were found to be negatively correlated with liver infiltrating CD4^+^ T cells and CD8^+^ T cells in all HCC patients. In addition, the expression of IFN-γ was lower in both cancerous and adjacent tissues in the GP73-positive patients compared with the GP73-negative patients, and IFN-γ was positively correlated with sGP73. Therefore, we hypothesize that these Foxp3^+^ Tregs may promote HCC progression by inhibiting T-cell proliferation and IFN-γ production in cancer patients. Meanwhile, Ki-67, a widely used marker associated with proliferation, is a nuclear antigen present exclusively in proliferating cells (33). In HCC, Ki-67 expression has been closely linked to the tumor growth rate (34) and identified as an independent prognostic indicator for worse outcomes (35). In the present study, GP73-positive HCC patients exhibited higher nuclear Ki-67 expression than GP73-negative patients. Furthermore, Ki-67 was positively correlated with sGP73 levels and Foxp3^+^ Treg cells. These findings indicate that increased sGP73 levels are associated with elevated levels of Foxp3^+^ Treg cells and reduced expression of CD8^+^ T cells. These correlations are further linked to decreased expression of IFN-γ and enhanced tumor viability, as defined by high Ki-67 expression. Taken together, these results suggest that the synergistic effect of low IFN-γ expression and reduced CD8^+^ T cells *in vivo* contributes to the progression of HCC. For example, GP73-positive patients exhibited tendencies toward higher mortality rate, higher rate of metastasis, higher AFP levels, more pronounced liver inflammation and damage, and higher likelihood of receiving second-line treatments compared with GP73-negative patients.

In summary, the present findings indicate that sGP73 may function as a diagnostic marker for HCC. In addition, the combined detection of sGP73 and sAFP could significantly enhance HCC diagnosis, providing an effective screening measure for high-risk populations. Furthermore, our data imply that GP73 is involved in HCC development through its interactions with the tumor microenvironment, by either directly or indirectly affecting the interactions between Treg cells and other immune effector cells. GP73 may influence the stability of these interactions or cytokine release. Due to our limited sample size, additional cases are required to validate the present findings. Moreover, it is crucial to emphasize that these hypotheses must undergo rigorous testing through additional experiments and studies. Researchers can utilize cell cultures, mouse models, and clinical case studies to delve deeper into the interactions and mechanisms involving GP73 and the microenvironment. Understanding these associations may provide insights into the biological processes of immune regulation and their potential applications in the treatment of HCC.

## Data Availability

The raw data supporting the conclusions of this article will be made available by the authors, without undue reservation.
